# Between Fact and Fabrication: How Visual Art Might Nurture Environmental Consciousness

**DOI:** 10.3389/fpsyg.2022.925843

**Published:** 2022-07-26

**Authors:** Rebecca Buening, Takuya Maeda, Kongmeng Liew, Eiji Aramaki

**Affiliations:** Social Computing Lab, Information Science Division, Graduate School of Science and Technology, Nara Institute of Science and Technology, Nara, Japan

**Keywords:** artistic visualization, artistic affectivization, data art, environmental consciousness, intersubjectivity, active caring

## Abstract

Previous studies have highlighted the communicative limitations of artistic visualizations, which are often too conceptual or interpretive to enhance public understanding of (and volition to act upon) scientific climate information. This seems to suggest a need for greater factuality/concreteness in artistic visualization projects, which may indeed be the case. However, in this paper, we synthesize insights from environmental psychology, the psychology of art, and intermediate disciplines like eco-aesthetics, to argue that artworks—defined by their counterfactual qualities—can be effective for stimulating elements of environmental consciousness. We also argue that different artworks may yield different effects depending on how they combine counter/factual strategies. In so doing, we assert that effective artistic perceptualization—here expressed as *affectivization*—exceeds the faithful translation of facts from one mode to another, and cannot be encapsulated in a single example of un/successful art.

## 1. Introduction

There is something of a contradiction in writing about artistic visualization: where common wisdom suggests that artworks can enhance audiences' receptiveness to climate science (as per Roberts, 2015), actual evaluations of specific works regard them as too conceptual to reliably do so (Kuchinskaya, 2018; Hahn and Berkers, 2021). Even documentary images are treated with suspicion (Zelizer, 2010; Lewandowsky and Whitmarsh, 2018). Rather than regard this as a fundamental limitation, we argue that the data perceptualization field lacks a coherent model explaining how visual art can promote target climate communication outcomes, which ones it is most suited to promote, and what features of art contribute to success in this task. Given this absence, practitioners apply the same criteria to artworks as they do to other forms of science communication, which is not wholly appropriate. In this paper, we begin the process of assembling such a model, arguing that:

1. artistic visualizations of climate data that primarily translate facts into visual forms are largely restricted to the communicative objective of fostering environmental literacy (Section 2.1);
2. art is defined by its counterfactual qualities, and so cannot straightforwardly convert climate data into perceptual forms; instead, it makes data perceptible through *affectivization*, allowing for the sampling of environmental dispositions (Section 2.2);
3. artworks that deliberately invoke factual and counterfactual strategies in mutually relevant ways (defined here as “data art”) may be able to support environmental consciousness (Section 3.1); and
4. there are myriad ways of combining these strategies across media, which may yield different outcomes, so the shortcomings of individual works do not necessarily reflect the absolute potential of all art (Section 3.2).

We apply the term “counterfactual” to communicative strategies that express what is not the case (Lea and Bradbery, 2020) or rest on an antecedent that is known to be false (Macintosh, 1995). This includes unrealized but possible scenarios, outright fictions, and figurative uses of language. By implication, we apply the term “factual” to communicative strategies that express what *is* the case or rest on an antecedent that is known to be true^1^. Though this argument recenters the role of truth in art, it does not privilege truth or regard it as an epistemological condition so much as a rhetorical convention. Indeed, all actual communication utilizes a combination of factual and counterfactual strategies (see, for instance, Minh-Ha, 1993; Zelizer, 2010; Dourish and Cruz, 2018 regarding the narrative conventions of documentary, journalistic photographs, and data, respectively). As per Elkins (1995) and Kemp (2005), we distinguish scientific and artistic communication by their differing expressive aims.

We draw on insights from diverse, interdisciplinary fields, including environmental psychology (Zelezny and Schultz, 2000), the psychology and anthropology of art (Ives and Pond, 1980; Gell, 1998; Dodell-Feder and Tamir, 2018), eco-aesthetics (Demos, 2013), ecocriticism/environmental humanities (Giles, 2014; Hegglund, 2020), and cognitive literary studies (Richardson, 2010; Oatley, 2017), making particular connections between models of environmental consciousness, explanations of the cognitive benefits of art and fiction, and literature on the interaction between reality and the imaginary. Some aspects of our argument are prefigured in works about aesthetic representation in journalism (Cramerotti, 2009; Zelizer, 2010). Our conclusion suggests that it is not only about how climate data is conveyed through “sound, light, and physical form,” as the current issue puts it, but how it is conveyed through various inflections of the imaginary. At the end of the paper, we present possible research directions that could further develop our argument.

## 2. Background

### 2.1. Artistic Visualization

Currently, the field of climate science communication operates on the “deficit model” (Hulme, 2009; Cook and Overpeck, 2019): the view that public inaction on climate change stems from a lack of information, and that scientific communication must educate in order to promote widespread behavioral change. In this model, communications present truth claims that should be judged based on their accuracy and reliability, ideally transferring information and fostering environmental literacy, or awareness of climate change consequences. Art within this paradigm is understood to facilitate factual communication by making scientific data more attention-grabbing, thought-provoking, and accessible to the wider public without changing its contents or aims. Too much interpretation causes experts to fret about the integrity of artistic communication, citing the risk that ambiguity poses to factual understanding, while too much visual simplification incites protests about the reductivism of art (Kallick-Wakker, 1994).

Though these critiques are valid, they constrain artistic visualizations within a narrow window of perceptualization akin to infographics. Take Laurie Frick's *What We Eat*^2^, an interactive interface contributed to Google Arts and Culture Lab's *Heartbeat of the Earth* series that visualizes the carbon emissions associated with users' daily food consumption (based on food emissions data from researchers at Tulane University and the University of Michigan). After mapping these meal and emission datasets onto each other to generate individual emission profiles, Frick uses a standardized figure “key” (listed in the “About” page)^3^ to transform these profiles into scaled and color-coded figures that are more intuitively legible and comparable. By allowing comparisons between different diets and people, the work successfully renders “the huge disparity between foods” and stimulates awareness about the environmental consequences of everyday food choices. However, it does not invite explorations beyond the data itself and prescribes only one correct interpretation of its contents. Thus, it may not facilitate the more-than informational objectives associated with art (Kieran, 1996, p. 343).

Contra the deficit model, scholars in various fields have acknowledged that obstacles to pro-environmental action exceed a lack of awareness (Van Liere and Dunlap, 1980; Dietz et al., 1998; Diekmann and Preisendörfer, 2003; Nyhan et al., 2022). Even where audiences can access credible information, this does not guarantee that they will accommodate it, especially within a contested media landscape (Singer, 2003; Carvalho and Burgess, 2005; Montes, 2018, p. 12–20) where audiences assess information experientially rather than analytically (Leiserowitz, 2006; Zelizer, 2010; Lehman et al., 2019). Some scholars question whether the concept of climate change can be expressed through “information” at all; Morton (2013) labels climate change as a “hyperobject,” something so vast as to be beyond comprehension. All of this points to “a fundamental disconnection between the methods (i.e., means) and objectives (i.e., ends) chosen by climate experts in the context of their interactions with publics” (Cook and Overpeck, 2019, p. 2).

Research on the disconnects between pro-environmental sentiment and action has yielded the multi-faceted concept of environmental consciousness (Van Liere and Dunlap, 1981; Stern et al., 1995; Dunlap and Van Liere, 2008), a sort of world-sense order that emerges at the intersection of cognitive processes, social dispositions and behaviors, and situational affordances. We base our definition of environmental consciousness on the model provided by Sánchez and Lafuente (2010) in their review of the literature, which outlines affective, dispositional, cognitive, and active dimensions of the concept. Art freed from the constraints of the deficit model could arguably operate within this model, contributing to a more capacious understanding of climate change.

### 2.2. Artistic Affectivization

Where artistic visualizations support the cognitive dimension of environmental consciousness, fine art movements support affective and dispositional objectives,“exchanging ‘matters of fact' for ‘matters of concern”' (Demos, 2013, p. 3, after Latour). Land art works, for instance, sought to decenter the capitalistic materialism of urban life by creating remote, larger-than-life earth monuments that could not be conveniently accessed or consumed, while subsequent environmental art like Agnes Denes's *Wheatfield—A Confrontation*^4^, a wheatfield planted on a Manhattan landfill to disrupt the perspective of the nearby financial district, launched interventions into cities. Contemporary ecological art explores alternative climate futures by undertaking green remediation projects.

The intrinsically counterfactual (or subjunctive; Zelizer, 2010, p. 12–15) nature of imagery, literature, and other figurative works provides a means to collectively imagine alternative realities. Since antiquity, art has been defined by its fabrication, its metaphorical (Oatley, 2012), dual ontological status, and thus by its displacement from the straightforwardly “real.” While this is partly based on the constitutive qualities of specific sensory media^5^, it also derives from the specific *use* of creative practices for subjunctive communication. For instance, photography and illustration can be used for primarily denotative (documentary) purposes, like photographic evidence and courtroom illustration, or for expressive, subversive, or other subjunctive purposes, as in most contemporary art^6^. Because the meaning of artworks exceeds what is literally depicted, genuinely artistic communication requires a kind of perspective-taking (see McConville, 2009, or Funk, 2021, in conversation with Ted Howell), conveying a frame of reference or “imaginative understanding” (Kieran, 1996) rather than discrete information. We call this artistic “affectivization,” after Salvatore et al. (2021)^7^: the “emergent” (or analogic; Ives and Pond, 1980; Seevinck, 2015) communication of subjective states that paves the way to intersubjectivity, or perspective-*sharing*. Gillespie and Cornish (2010) assert that intersubjectivity is the objective of all human communication, but that it often exceeds notions of shared understanding or agreement (c.f. Maul et al., 2019), involving collaborative and continual reorientation of the self to one's interlocutors. Thus, objectifying inner worlds through art may allow humans to orient our respective subjective stances, our ambitions and plans, and our moral sensibilities and expectations (Dewey, 1958; Bourdieu and Nice, 1984; Kieran, 1996).

Artists facilitate intersubjectivity in multiple ways, and at various registers. Most fundamentally, they instate a shared cultural logic/vocabulary that can be used to broach difficult topics (see, for example, Funk, 2021 in conversation with Amy Brady) or assert counter-narratives to those enshrined in history. One example is the work of Allison Janae Hamilton, who countervails the white-washing of climate narratives by foregrounding southern African American folklore traditions in her environmental imaginaries^8^. Over time, the practice of such art solidifies culture, rendering the counterfactual real by training sensibilities (see Bourdieu and Nice, 1984), inscribing identity, and building capacities. Consider the *Trees for Life* project undertaken by Earth Art Studio and Ethiopia's Rural Organization for the Betterment of Agro-Pastoralists, which seeks to revitalize native Oromo forest stewardship practices by communally planting a lion-shaped tree nursery (Anania, 2021). These strategies foster feelings of belonging, modify individuals' self-perception/construal, and nurture self-efficacy and empowerment. Indeed, findings from the field of art education show that plastic and performing arts can facilitate transformative learning that cultivates a sense of agency and environmental responsibility amongst students (Häggström, 2019), while findings from cognitive literary studies demonstrate that figurative literature empowers readers to undertake self-directed personal change (Oatley, 2017).

Some artists strive for emotional communication, or the direct depiction of subjective states that viewers may access through cognitive mindreading and stylistic analysis. Such is the case for works of literary fiction, which have been shown to improve readers' Theory of Mind, empathy, and social cognition over time (Oatley, 2012; Kidd and Castano, 2013; Dodell-Feder and Tamir, 2018), but also of plastic or performance arts that foreground a specific vantage point. Take Tim Gaudreau's *Self-Portrait as Revealed by Trash*^9^, wherein the artist photographed every piece of trash he created over 365 days to represent his broader life. Other artworks present scenes that suggest a larger narrative, weave counterfactuals into a distinct world that audiences can imaginatively explore (Kieran, 1996), or even destabilize ordinary perception in ways that trigger metacognition and self-world orientation (Nikolajeva, 2016). In each case, viewers are made to position themselves within/relative to the work. This occurs most frequently in long-form storytelling like novels, film, and video games, though it can also manifest in (eco-)surrealist art like that of Josh Keyes^10^, op-art, or any work supplemented with informational content like artist statements, museum placards, or audio guides. James Turrell's light-based artworks are an excellent example of induced metacognition (and of perspective-taking without substantial information-transfer): in his *Ganzfelds* series^11^, Turrell manipulates lights and colors to create an illusional space wherein visitors lose their depth perception, simulating the experience of white-out and thus provoking reflection on perception itself^12^.

Insofar as artworks provide access to empowered, belonging, or empathic attitudes, they may nurture what Geller (1995) called an “active caring” person state: an agentic, pro-social disposition that correlates with greater environmental action. The presence of environmental self-efficacy and other empowering states, for instance, can yield more optimistic environmental targets and more pro-environmental consumer behavior (Sawitri et al., 2015; Derdowski et al., 2020). With respect to belonging, researchers acknowledge differential engagement in problems that are presented as proximate or distant to the subject (Uzzell, 2000; García-Mira et al., 2005; Ballantyne et al., 2018), provoking explorations for how to incorporate local-level subject matter into climate change visualizations (Burch et al., 2009; Schroth et al., 2014; Ballantyne et al., 2018). Meanwhile, altruistic and “biospheric” understandings of environmental consciousness (typically expressed as environmental concern; Stern and Dietz, 1994; Schultz, 2000) directly implicate empathy and Theory of Mind in pro-environmental behavior—either as it is extended toward other people or to non-human entities, respectively. Perhaps periodically assuming an active caring person state, even if vicarious, can eventually support more enduring personal moral norms, consistent with Schwartz's (1977) Norm-Activation Model of environmental concern (p. 231–241).

Of course, not all artworks succeed in rendering these states, and those that do are only temporarily effective (Schneider-Mayerson et al., 2020). Consequently, artworks should form one part in a larger curriculum of attention. More urgently, while affectivization *can* be used for pro-social ends, without adequate factual bases it can also make it easier to dismiss uncomfortable realities (Zelizer, 2010, p. 15–23), stoke flames of misinformation (Lowe et al., 2006; Funk, 2021; Salvatore et al., 2021), manipulate people's perceptions (Gell, 1992), or concretize problematic worldviews through propaganda. It is essential, then, that artistic representation engage with notions of artistic responsibility and provide a range of perspectives that audience members can sample.

## 3. Discussion

### 3.1. Proposed Scope of Data Art

Pursued separately, both artistic visualization and artistic affectivization have important limitations. Where the former eschews ambiguity and overlooks the background of experiences and perspectives that inflect reasoning (Seevinck, 2015), the latter can be (ab)used to transmit empty or problematic fictions. Moreover, both forms of communication struggle to prompt/sustain subsequent action (Zelizer, 2010, p. 20). But just as actual communication uses both factual and counterfactual strategies, many effective communications deliberately engage in both information transfer and perspective-sampling (or visualization *and* affectivization). Indeed, the careful calibration of factual information transfer and counterfactual perspective sampling afford distinct communicative opportunities that exceed the potential of either strategy alone, serving to locate foreign content within understandable frames of reference (for instance, through association with familiar forms; Kallick-Wakker, 1994; Zelizer, 2010) or to make established frames of reference seem foreign and strange. Take, for instance, Olafur Eliasson's *Ice Watch*, which migrates fragments of arctic glaciers (a real artifact) to public squares in Paris and London in order to demonstrate the rate at which they melt in warmer temperatures (a counterfactual, but impending, scenario). The artwork draws on the visual repertoire of monumental/memorial art, given the size and location of the objects and the theme of loss. Moreover, the work actualizes the insights of climate forecasting data, making it tangible and literally present. Together, these strategies confront viewers with a palpable, historic tragedy.

Data art might take a page from longstanding discussions about the factual and counterfactual dimensions of photography, wherein Barthes described a “third meaning” beyond “literal and figurative meaning” (Zelizer, 2010). Where Barthes and Howard (1981) called this the “punctum” and Zelizer (2010) calls it “voice,” both accounts emphasize how multi-valent communication exceeds cognitive-perceptual or affective-dispositional dimensions, stimulating active attention. Zelizer (2010) elaborates, “While denotation grounds the image in reality, and connotation carries the meaning of an image across a set of possible associations, voice orients to the ways in which an image travels *via* these associations to other contexts, where it can be used by other people, seen through other images, and activated for other aims” (p. 13). Insofar as this third meaning also abuts the psychoanalytic notion of “thirdness” described by Salvatore et al. (2021)—the “third-as-other” who limits one's own totalizing interest (p. 19) and cannot be encompassed by denotative/connotative signification—it can trigger some momentary confrontation with the *actual* beyond the given counter/factuals. This is a potential worthy of future study.

### 3.2. Data Art Strategies

The deliberate interpolation of factual and counterfactual strategies reveals the potential fruitfulness and *diversity* of ambiguous states, which may also be characterized as “ontological instability” (or “*weirdness*”; Montes, 2018), “limit situations” (Tsang, 1998), or “liminal states” (Turner and Abrahams, 2017). It also reveals the means by which artworks can overcome or *induce* such states—for instance, by aligning or opposing factual and counterfactual propositions to stimulate consonant *or* dissonant information-transferring and perspective-sampling processes. Some research suggests that cognitive dissonance may support the actualization of empathy as altruistic action in ways that more positive effects cannot (Schneider-Mayerson, 2018; Funk, 2021), namely by confronting subjects with their contradictory thoughts/behaviors in ways that are so uncomfortable as to provoke self-adjustment (Harmon-Jones et al., 2003). This could help to foreground problems like individuals' tendency to downplay the significance of environmental problems in which they are personally involved (García-Mira et al., 2005) or “the natural tendency of social memory [...] to suppress what is not meaningful or intuitively satisfying” (Zelizer, 2010, p. 5, after Michael Griffin). By preventing viewers from preemptively stabilizing the meaning of what they see—that is, by *sustaining* or even magnifying ambiguity—dissonant counter/factual strategies may also be uniquely suited to evoking hyperobjects or the “slow violence” of human–environment relations (Wong, 2017; Ito, 2021, p. 132–136), which cannot be “made sensible.”

It is essential, though, that the given counter/factual strategies do not simply coincide, as a lack of meaningful relation between them could yield the (undesirable) ambiguous state of confusion. Instead, combinations and permutations of counter/factual strategies should be explored early on, possibly as a constitutive component of artistic genre. This would pave the way for studies that identify genre-specific blends of fact and counterfact and their relation to different kinds of ambiguity/liminality, or studies that examine whether different media afford different strategies and liminal states (see, for instance, McLuhan, 1994, or even Ives and Pond, 1980, p. 337–339). We might expect synthetic art (Cosne et al., 2020), which blurs the boundaries of factuality and counterfactuality, to render our relationship with the actual world differently than other artforms. If so, how might we mobilize these affordances to communicate specific climate change perspectives (Stern, 2000, p. 420–421)?

Answers to these questions could elucidate a more formal and testable conceptual model that could help artists (and other actors) to plan their communications. At present, they suffice to dismantle the notion that “art” is a homogeneous category whose objects are commensurable with each other. Though we have attempted to provide some theoretical basis for comparison, it can only be partial and contingent, requiring further appraisal of works according to their particular qualities and contexts.

## 4. Conclusion

In this paper, we have drawn upon psychological models of environmental consciousness and literature on the cognitive benefits of art and fiction to illustrate how various combinations of factual and counterfactual strategies might align—or create tension between—constituent dimensions of environmental consciousness (as distinct from other objectives of climate communication), thereby creating unique routes to potential environmental action. Moreover, we have outlined differences between three kinds of artistic perceptualization: artistic visualization, artistic affectivization, and approaches that combine the two (data art). Where the first informs and the second moves (as per Aesthetic Trinity Theory; Konečni, 2011), the last, ideally, *triggers*. Together, our assertions promote a concept of data art that exceeds mere translation and implicates material forms within conceptual processes.

The perspectives presented here could be developed in several ways, both by extending the inquiry into new fields and by following it into new theoretical directions. For example, neuroaesthetic studies could supplement phenomenological research and psychological measures of environmental consciousness to empirically map connections between constructs. Alternatively, research could expand on specific person/liminal states, such as by exploring the connections between optimism, active caring, and intersubjectivity. On the philosophical side, researchers could investigate the possibility of an environmental morality within environmental consciousness, insofar as it conceptually extends empathic thinking and agency to non-human, non-animal others. This includes studies of artworks that include non-human animals within their audience, such as Jim Nollman's musical performances. Politically-oriented studies could examine the implications of this argument within ongoing discussions of rationalizing/affectivizing the public sphere (Zelizer, 2010, p. 7; Salvatore et al., 2021). Finally, researchers could expand beyond data art and fine art methodologies altogether to investigate the intersection of participatory/performative art interventions and (bio-)engineering, as well as daily practices of aesthetic perception.

## Author Contributions

RB undertook the literature review, developed the argument, and wrote up the conceptual portions of the paper. TM helped to refine and shorten this argument, sourced and analyzed the examples, and wrote up the illustrative portions of the paper. KL corrected the psychological aspects of the argument and helped to pare down the material. EA managed the project's development. All authors agree to be accountable for the content of the work. All authors contributed to the article and approved the submitted version.

## Conflict of Interest

The authors declare that the research was conducted in the absence of any commercial or financial relationships that could be construed as a potential conflict of interest.

## Publisher's Note

All claims expressed in this article are solely those of the authors and do not necessarily represent those of their affiliated organizations, or those of the publisher, the editors and the reviewers. Any product that may be evaluated in this article, or claim that may be made by its manufacturer, is not guaranteed or endorsed by the publisher.

## Footnotes

^1^See Table S1 for additional information about our choice of terms.

^2^ https://experiments.withgoogle.com/what-we-eat

^3^Blue ring = male, orange ring = female, warm colors/larger circles = higher emission foods/outputs, cool colors/smaller circles = lower emission foods/outputs, etc.

^4^ http://www.agnesdenesstudio.com/works7.html

^5^Where a photograph departs from the real by isolating information and suspending time (Zelizer, 2010, following Terence Wright), an illustration departs from the real *via* the artist's mediation, reflecting the artist's interpretation of something as much as the thing itself.

^6^Consider the fairytale-esque work of Sally Mann (photographer) and Yoshitaka Amano (illustrator), or the politically irreverent work of Andres Serrano (photographer) and most political cartoonists.

^7^Though Salvatore et al. (2021) feature a negative instance of affectivization—the blind affectivization of the public sphere—they concede that it is a subconscious, sense-making logic that underwrites cognition, shapes experience, and links individuals and socio-cultural contexts through the provision of “semiotic capital,” or intangible symbolic resources (p. 15–18).

^8^See, for instance, *A Romance of Paradise?*: https://www.allisonjanaehamilton.com/a-romance-of-paradise. For non-environmental art examples, see Thenmozhi Soundararajan's work: https://www.theguardian.com/world/2015/mar/12/thenmozhi-soundararajan-dalit-women-art-and-activism.

^9^ http://timgaudreau.com/2018/02/13/self-portrait-variation-1-2006/

^10^ https://stephenkellycreative.wordpress.com/2012/11/15/everythings-gone-green-the-eco-surrealism-of-josh-keyes/

^11^ https://jamesturrell.com/work/type/ganzfeld/

^12^Prominent examples outside of environmental art include Velasquez's *Las Meninas* painting, which presents a complex web of sight lines between figures and reflections that incorporates the viewer as an object within the painting, and arresting non-art images like that of Neda Agha-Soltan—an Iranian philosophy student slain in the country's 2009 election protests—looking at the camera plaintively as she dies (quoting a trade paper, Zelizer, 2010 explains that the “viewer cannot help but imagine being there”; p. 9).

## References

[B1] Anania B (2021). In Ethiopia, artists protect the environment through “plant graffiti”. *Hyperallergic*. Available online at: https://hyperallergic.com/692602/ethiopia-lion-land-art-climate-change/ (accessed April 21, 2022).

[B2] Ballantyne A. G. Glaas E. Neset T.-S. S. Wibeck V. (2018). Localizing climate change: Nordic homeowners' interpretations of visual representations for climate adaptation. Environ. Commun. 12, 638–652. 10.1080/17524032.2017.1412997

[B3] Barthes R (1981). Camera Lucida: Reflections on Photography. New York, NY: Hill and Wang.

[B4] Bourdieu P. Nice R. (1984). Distinction: A Social Critique of the Judgement of Taste. Cambridge, MA: Harvard University Press.

[B5] Burch S. Shaw A. Sheppard S. Flanders D. (2009). “Climate change visualization: using 3D imagery of local places to build capacity and inform policy,” in State of Climate Visualization (Report 09:04), eds T. Neset, J. Johansson, and B. Linnér (Norrköping: The Center for Climate Science and Policy Research), 65–73. Available online at: http://www.cspr.se/publications (accessed April 21, 2022).

[B6] Carvalho A. Burgess J. (2005). Cultural circuits of climate change in U.K. broadsheet newspapers, 1985–2003. Risk Anal. 25, 1457–1469. 10.1111/j.1539-6924.2005.00692.x 16506975

[B7] Cook B. R. Overpeck J. T. (2019). Relationship-building between climate scientists and publics as an alternative to information transfer. Wiley Interdisc. Rev.: Climate Change 10:e570. 10.1002/wcc.570

[B8] Cosne G. Juraver A. Teng M. Schmidt V. Vardanyan V. T. Luccioni A.. (2020). Using simulated data to generate images of climate change. arXiv [Preprint]. arXiv: 2001.09531. 10.48550/arXiv.2001.09531

[B9] Cramerotti A (2009). Aesthetic Journalism: How to Inform Without Informing. Bristol; Chicago, IL: Intellect Books.

[B10] Demos T. J (2013). Contemporary art and the politics of ecology: an introduction. Third Text 27, 1–9. 10.1080/09528822.2013.753187

[B11] Derdowski L. A. Grahn Å. H. Hansen H. Skeiseid H. (2020). The new ecological paradigm, pro-environmental behaviour, and the moderating effects of locus of control and self-construal. Sustainability 12:7728. 10.3390/su12187728

[B12] Dewey J (1958). “Art and civilization,” in Art as Experience, ed J. Dewey (New York, NY: Capricorn Books), 326–349.

[B13] Diekmann A. Preisendörfer P. (2003). Green and greenback: the behavioral effects of environmental attitudes in low-cost and high-cost situations. Rational. Soc. 15, 441–472. 10.1177/1043463103154002

[B14] Dietz T. Stern P. C. Guagnano G. A. (1998). Social structural and social psychological bases of environmental concern. Environ. Behav. 30, 450–471. 10.1177/00139165980300040222763047

[B15] Dodell-Feder D. Tamir D. I. (2018). Fiction reading has a small positive impact on social cognition: a meta-analysis. J. Exp. Psychol. 147, 1713–1727. 10.1037/xge0000395 29481102

[B16] Dourish P. Cruz E. G. (2018). Datafication and data fiction: narrating data and narrating with data. Big Data Soc. 5. 10.1177/2053951718784083

[B17] Dunlap R. E. Van Liere K. D. (2008). The “new environmental paradigm”. J. Environ. Educ. 40, 19–28. 10.3200/JOEE.40.1.19-28

[B18] Elkins J (1995). Art history and images that are not art. Art Bull. 77, 553–571. 10.2307/3046136

[B19] Funk A (2021). Can climate fiction writers reach people in ways that scientists can't? *Smithsonian Magazine*. Available online at: https://www.smithsonian mag.com/innovation/can-climate-fiction-writers-reach-people-ways-scientists -cant-180977714/ (accessed April 21, 2022).

[B20] García-Mira R. Real J. E. Romay J. (2005). Temporal and spatial dimensions in the perception of environmental problems: an investigation of the concept of environmental hyperopia. Int. J. Psychol. 40, 5–10. 10.1080/00207590444000078

[B21] Gell A (1992). “The technology of enchantment and the enchantment of technology,” in Anthropology, Art and Aesthetics, eds J. Coote and A. Shelton (New York, NY: Clarendon Press), 40–63.

[B22] Gell A (1998). Art and Agency: An Anthropological Theory. Oxford: Clarendon Press.

[B23] Geller E. S (1995). Integrating behaviorism and humanism for environmental protection. J. Soc. Issues 51, 179–195. 10.1111/j.1540-4560.1995.tb01354.x

[B24] Giles J. M (2014). Can the sublime be postcolonial? Aesthetics, politics, and environment in Amitav Ghosh's *The Hungry Tide*. Camb. J. Postcol. Liter. Inq. 1, 223–242. 10.1017/pli.2014.18

[B25] Gillespie A. Cornish F. (2010). Intersubjectivity: towards a dialogical analysis. J. Theory Soc. Behav. 40, 19–46. 10.1111/j.1468-5914.2009.00419.x

[B26] Häggström M (2019). “Students being transformed into trees: inverted anthropomorphization in order to enhance connectedness to natural environments and plants,” in Art, Theory and Practice in the Anthropocene, ed J. Reiss (Wilmington, DE: Vernon Press), 137–153.

[B27] Hahn U. Berkers P. (2021). Visualizing climate change: an exploratory study of the effectiveness of artistic information visualizations. World Art 11, 95–119. 10.1080/21500894.2020.1769718

[B28] Harmon-Jones E. Peterson H. Vaughn K. (2003). The dissonance-inducing effects of an inconsistency between experienced empathy and knowledge of past failures to help: support for the action-based model of dissonance. Basic Appl. Soc. Psychol. 25, 69–78. 10.1207/S15324834BASP2501_5

[B29] Hegglund J (2020). “Unnatural narratology and weird realism in Jeff VanderMeer's Annihilation,” in Environment and Narrative: New Directions in Econarratology, eds E. James and E. Morel (Columbus, OH: Ohio State University Press), 27–44.

[B30] Hulme M (2009). Why We Disagree About Climate Change: Understanding Controversy, Inaction and Opportunity. New York, NY: Cambridge University Press. 10.1017/CBO9780511841200

[B31] Ito A (2021). Translating slow violence: the use of environmental data in art as un-forecasting. AM J. Art Media Stud. 25, 39–48. 10.25038/am.v0i25.459

[B32] Ives W. H. Pond J. B. (1980). The arts and cognitive development. High School J. 63, 335–340. Available online at: https://www.jstor.org/stable/40365007 (accessed April 21, 2022).

[B33] Kallick-Wakker I (1994). Science icons: the visualization of scientific truths. Leonardo 27, 309–315. 10.2307/1576006

[B34] Kemp M (2005). From science in art to the art of science. Nature 434, 308–309. 10.1038/434308a 15772646

[B35] Kidd D. C. Castano E. (2013). Reading literary fiction improves Theory of Mind. Science 342, 377–380. 10.1126/science.1239918 24091705

[B36] Kieran M (1996). Art, imagination, and the cultivation of morals. J. Aesthet. Art Critic. 54, 337–351. 10.2307/431916

[B37] Konečni V. J (2011). Aesthetic trinity theory and the sublime. Philos. Today 55, 64–73. 10.5840/philtoday20115516226442338

[B38] Kuchinskaya O (2018). Connecting the dots: public engagement with environmental data. Environ. Commun. 12, 495–506. 10.1080/17524032.2017.1289106

[B39] Lea D. Bradbery J. (2020). “counterfactual, adj.,” in Oxford Advanced Learner's Dictionary (10th edition), eds D. Lea and J. Bradbery (Oxford: Oxford University Press). Available online at: https://www.oxfordlearnersdictionaries.com/definition/english/counterfactual_1 (accessed May 26, 2022).

[B40] Lehman B. Thompson J. L. Davis S. K. Carlson J. M. (2019). Affective images of climate change. Front. Psychol. 10:960. 10.3389/fpsyg.2019.00960 31156493 PMC6529642

[B41] Leiserowitz A (2006). Climate change risk perception and policy preferences: the role of affect, imagery, and values. Clim. Change 77, 45–72. 10.1007/s10584-006-9059-9

[B42] Lewandowsky S. Whitmarsh L. (2018). Climate communication for biologists: when a picture can tell a thousand words. PLoS Biol. 16:e2006004. 10.1371/journal.pbio.2006004 30300345 PMC6177125

[B43] Lowe T. Brown K. Dessai S. de França Doria M. Haynes K. Vincent K. (2006). Does tomorrow ever come? Disaster narrative and public perceptions of climate change. Publ. Understand. Sci. 15, 435–457. 10.1177/0963662506063796

[B44] Macintosh J (1995). “counterfactuals, n.,” in The Oxford Companion to Philosophy, ed T. Honderich (New York, NY: Oxford University Press), 169. Available online at: https://www.oxfordreference.com/view/10.1093/oi/authority.20110803095642948 (accessed May 26, 2022).

[B45] Maul A. Mari L. Wilson M. (2019). Intersubjectivity of measurement across the sciences. Measurement 131, 764–770. 10.1016/j.measurement.2018.08.068

[B46] McConville D (2009). “Visualizing worldviews: shifting perspectives on climate change,” in State of Climate Visualization (Report 09:04), eds T. Neset, J. Johansson, and B. Linnér (Norrköping: The Center for Climate Science and Policy Research), 9–18. Available online at: http://www.cspr.se/publications (accessed April 21, 2022).

[B47] McLuhan M (1994). “The medium is the message,” in Understanding Media: The Extensions of Man, eds M. McLuhan and L. H. Lapham (Cambridge, MA: The MIT Press), 7–21.

[B48] Minha-Ha T. T (1993). “The totalizing quest of meaning,” in Theorizing Documentary, ed M. Renov (New York, NY: Routledge), 90–107. 10.4324/9780203873083

[B49] Montes A. E (2018). Sublime annihilation and the Weird: *an aesthetic for times of anthropocene indeterminacy* (Master's thesis). Faculty of Humanities; University of Amsterdam, Amsterdam, Netherlands.

[B50] Morton T (2013). Hyperobjects: Philosophy and Ecology after the End of the World. Minneapolis, MN: University of Minnesota Press.

[B51] Nikolajeva M (2016). Navigating fiction: cognitive-affective engagement with place in children's literature. *Breac: A Digital Journal of Irish Studies*. Available online at: https://breac.nd.edu/articles/navigating-fiction-cognitive-affective-engagement-with-place-in-childrens-literature/ (accessed April 21, 2022).

[B52] Nyhan B. Porter E. Wood T. J. (2022). Time and skeptical opinion content erode the effects of science coverage on climate beliefs and attitudes. PNAS 119:e2122069119. 10.1073/pnas.2122069119 35727983 PMC9245713

[B53] Oatley K (2012). The cognitive science of fiction. Wiley Interdisc. Rev.: Cognitive Science 3, 425–430. 10.1002/wcs.1185 26301527

[B54] Oatley K (2017). “On truth and fiction,” in Cognitive Literary Science: Dialogues between Literature and Cognition, eds M. Burke and E. T. Troscianko (New York, NY: Oxford University Press), 259–278. 10.1093/acprof:oso/9780190496869.003.0014

[B55] Richardson A (2010). The Neural Sublime: Cognitive Theories and Romantic Texts. Baltimore, MD: Johns Hopkins University Press.

[B56] Roberts L (2015). Living data: how art helps us all understand climate change. *The Conversation*. Available online at: https://theconversation.com/living-data-how-art-helps-us-all-understand-climate-change-36890 (accessed April 21, 2022).

[B57] Salvatore S. Picione R. D. L Vincenzo B. Mannino G. Langher V. Pergola F.. (2021). The affectivization of the public sphere: the contribution of psychoanalysis in understanding and counteracting the current crisis scenarios. Subject Action Soc.: Psychoanal. Stud. Pract. 1, 3–30. 10.32111/SAS.2021.1.1.2

[B58] Sánchez M. J. Lafuente R. (2010). Defining and measuring environmental consciousness. Rev. Int. Sociol. 68, 731–755. 10.3989/ris.2008.11.0328837691

[B59] Sawitri D. R. Hadiyanto H. Hadi S. P. (2015). Pro-environmental behavior from a socialcognitive theory perspective. Proc. Environ. Sci. 23, 27–33. 10.1016/j.proenv.2015.01.005

[B60] Schneider-Mayerson M (2018). The influence of climate fiction: an empirical survey of readers. Environ. Human. 10, 473–500. 10.1215/22011919-7156848

[B61] Schneider-Mayerson M. Gustafson A. Leiserowitz A. Goldberg M. H. Rosenthal S. A. Ballew M. T. (2020). Environmental literature as persuasion: an experimental test of the effects of reading climate fiction. Environ. Commun. 10.1080/17524032.2020.1814377

[B62] Schroth O. Angel J. Sheppard S. Dulic A. (2014). Visual climate change communication: from iconography to locally framed 3D visualization. Environ. Commun. 8, 413–432. 10.1080/17524032.2014.906478

[B63] Schultz P. W (2000). Empathizing with nature: the effects of perspective taking on concern for environmental issues. J. Soc. Issues 56, 391–406. 10.1111/0022-4537.00174

[B64] Schwartz S. H (1977). “Normative influences on altruism,” in Advances in Experimental Social Psychology, ed L. Berkowitz (Cambridge, MA: Academic Press), Vol. 10, 221–279. 10.1016/S0065-2601(08)60358-5

[B65] Seevinck J (2015). Emergence in interactive artistic visualization. Int. J. Softw. Eng. Knowl. Eng. 25, 201–230. 10.1142/S0218194015400070

[B66] Singer J. B (2003). Who are these guys? The online challenge to the notion of journalistic professionalism. Journalism 4, 139–163. 10.1177/146488490342001

[B67] Stern P. C (2000). Toward a coherent theory of environmentally significant behavior. J. Soc. Issues 56, 407–424. 10.1111/0022-4537.00175

[B68] Stern P. C. Dietz T. (1994). The value basis of environmental concern. J. Soc. Issues 50, 65–84. 10.1111/j.1540-4560.1994.tb02420.x

[B69] Stern P. C. Dietz T. Guagnano G. A. (1995). The new ecological paradigm in social-psychological context. Environ. Behav. 27, 723–743. 10.1177/001391659527600135393503

[B70] Tsang L.-C (1998). The Sublime: Groundwork Towards a Theory. Rochester, NY; Suffolk: University of Rochester Press.

[B71] Turner V. Abrahams R. D. (2017). The Ritual Process: Structure and Anti-Structure. Abingdon; New York, NY: Routledge. 10.4324/9781315134666

[B72] Uzzell D. L (2000). The psycho-spatial dimension of global environmental problems. J. Environ. Psychol. 20, 307–318. 10.1006/jevp.2000.0175

[B73] Van Liere K. D. Dunlap R. E. (1980). The social bases of environmental concern: a review of hypotheses, explanations and empirical evidence. Publ. Opin. Quart. 44, 181–197. 10.1086/268583

[B74] Van Liere K. D. Dunlap R. E. (1981). Environmental concern: does it make a difference how it's measured? Environ. Behav. 13, 651–676. 10.1177/0013916581136001

[B75] Wong A. K (2017). Beyond anthropocentric futurism: visualizing air pollution and waste in post-Olympic Beijing. Concentric: Liter. Cult. Stud. 43, 119–143. 10.6240/concentric.lit.2017.43.1.0722096505

[B76] Zelezny L. C. Schultz P. W. (2000). Promoting environmentalism. J. Soc. Issues 56, 365–371. 10.1111/0022-4537.00172

[B77] Zelizer B (2010). “Journalism, memory, and the voice of the visual,” in About to Die: How News Images Move the Public, ed B. Zelizer (New York, NY: Oxford University Press), 1–27.

