# Supplementary material for: Between Fact and Fabrication: How Visual Art Might Nurture Environmental Consciousness
Source: Front Psychol. 2022 Jul 26;13:925843. doi: 10.3389/fpsyg.2022.925843 (PMC9360767; doi:10.3389/fpsyg.2022.925843)
Supplement: Supplementary file 1 [file Table_1.pdf]

Supplementary Material

1 SUPPLEMENTARY TABLE

**Perceptualization type**	**Visualization**	**Multi-valent** (Data art)	**Affectivization**	
**Communicative mode**	Information-transferring	Multi-valent	Perspective-sharing (long-term); Perspective-sampling (short-term)	
**Communicative objective**	Environmental literacy<br><br>Awareness of the causes, processes, and consequences of environmental phenomena	Environmental consciousness<br><br>A sort of world-sense order that emerges at the intersection of cognitive processes, social dispositions and behaviors, and situational affordances	Environmental disposition<br><br>(Temporary or vicarious) assumption of pro-environmental attitudes, perspectives, or “person states” (including self-efficacy, optimism, belonging, etc.)	
**Dominant strategy**	Factual<br><br>Communicative strategies that express what is the case or rest on an antecedent that is known to be true	Varies	Counterfactual<br><br>Communicative strategies that express what is not the case or rest on an antecedent that is known to be false	
Applied to strictly falsifiable content	“Factual” content; realized scenarios<br><br>*“Michelle Obama is a mother”*	Varies	“Fictional” (fictive, fabricated) content<br><br>*“Minnie Mouse is a mother”*	
Applied to potentially falsifiable content	“Assumed” content; likely realized but unsubstantiated scenarios<br><br>*“Mom makes the best food”*	Varies	“Speculative” content; unrealized but possible scenarios<br><br>*“If I were a mother. . . ”*	
Applied to non-falsifiable linguistic devices/frames	“Naturalistic” (literal) uses of language<br><br>*“We were born on earth”*	Varies	“Figurative” (metaphorical) uses of language<br><br>*“The earth is our mother”*	
**Example**	Infographics; educational media	Proposed scoped of data art	Environmental art	

Table S1. Explanation/organization of terms and concepts
